# Endoscopic Palliative Treatment for Duodenal and Biliary Obstruction in Advanced Periampullary Malignancy

**DOI:** 10.7759/cureus.64912

**Published:** 2024-07-19

**Authors:** Anusha Gupta, Vishal Padwale, Vijendra Kirnake, Aishwarya Gupta, Sourav Chaturvedi

**Affiliations:** 1 Department of Gastroenterology, Datta Meghe Institute of Higher Education and Research, Wardha, IND; 2 Department of Obstetrics and Gynaecology, Datta Meghe Institute of Higher Education and Research, Wardha, IND; 3 Department of Cardiology, Max Super Speciality Hospital, Delhi, IND

**Keywords:** duodenal stenting, ercp, endoscopic palliation, biliary obstruction, duodenal obstruction, periampullary malignancy

## Abstract

Unresectable periampullary malignancies can lead to concomitant duodenal and biliary obstructions, significantly affecting patient quality of life. Effective palliation of these obstructions is crucial for symptom management and improving patient outcomes. Endoscopic techniques provide a minimally invasive approach to address these complications. This report presents a case where endoscopy was successfully used to palliate both duodenal and biliary obstructions in a patient with advanced periampullary malignancy. Endoscopic retrograde cholangiopancreatography was attempted to relieve the biliary obstruction caused by periampullary malignancy; however, the procedure was subsequently abandoned and the patient ultimately underwent percutaneous transhepatic biliary drainage. Furthermore, the use of an endoscope for duodenal stenting to restore gastrointestinal continuity was done. The patient experienced significant symptomatic relief and improved quality of life post-procedure. This case underscores the utility of endoscopic interventions in managing complex obstructions due to advanced malignancies.

## Introduction

Periampullary adenocarcinoma is a malignancy of the pancreatic head, ampulla, and distal common bile duct, and 80% of these tumors need palliative treatment [1]. Complications related to biliary and duodenal compression caused by the cancer and tumor invasion occur in 80% and 20% of cases, respectively [1,2]. Advanced pancreaticobiliary, gastroduodenal, and metastatic malignancies that are unresectable can result in simultaneous biliary and duodenal obstruction. Biliary obstruction is observed in 51%-72% of cases with advanced pancreaticobiliary cancers [1,2], while duodenal obstruction rates have also increased to 38% due to improvements in oncological treatments, resulting in longer patient survival.

Jaundice can lead to pruritis, which is disabling with malnourishment and intractable vomiting caused by duodenal obstruction [3]. The development of endo-prosthetic stents has changed the management of these patients, whereas, in the past, an open surgical palliative bypass was the only available option. The success rate is greater than 90% for duodenal and biliary stent placement with low morbidity [3,4].

## Case presentation

A 60-year-old male patient with no comorbidities presented with complaints of yellowish discoloration of eyes and skin and recurrent vomiting along with loss of weight and appetite for one month. The patient had a previously diagnosed case of periampullary carcinoma (histologically proven moderately differentiated adenocarcinoma of the distal common bile duct) since 2017 but was not taking any treatment for the same. On examination, the patient was icteric. Per abdomen examination, the abdomen was soft and non-tender and there was no palpable mass on the abdomen or dilated veins. On abdominal auscultation, bowel sound was increased.

Routine investigations were done that showed hemoglobin of 13.2 g/dL, white blood cell (WBC) count of 15,000/mm^3^, platelet count of 2.67 lakhs/mm^3^, total bilirubin of 19.2 mg/dL, conjugated bilirubin of 15.2 mg/dL, serum alanine transaminase of 290 IU/L, serum aspartate transaminase of 210 IU/L, serum alkaline phosphatase of 900 IU/L, serum cancer antigen 19-9 of 540 IU/L, serum urea of 20 mg/dL, serum creatinine of 1.2 mg/dL, serum sodium of 132 mEq/L, and serum potassium of 3.0 mEq/L. The patient’s coagulation profile was also deranged with an international normalized ratio of 2.1 and an activated plasma thromboplastin time of 56 seconds. An X-ray of the erect abdomen showed dilated bowel loops without any signs of perforation.

A nasogastric tube was inserted and the patient was kept nil by mouth with continuous nasogastric aspiration to relieve the obstruction. The patient was started on higher antibiotics (piperacillin-tazobactam and metronidazole) for a raised WBC count. Additionally, the patient was given a plasma transfusion because of a deranged coagulation profile.

**Figure 1:**
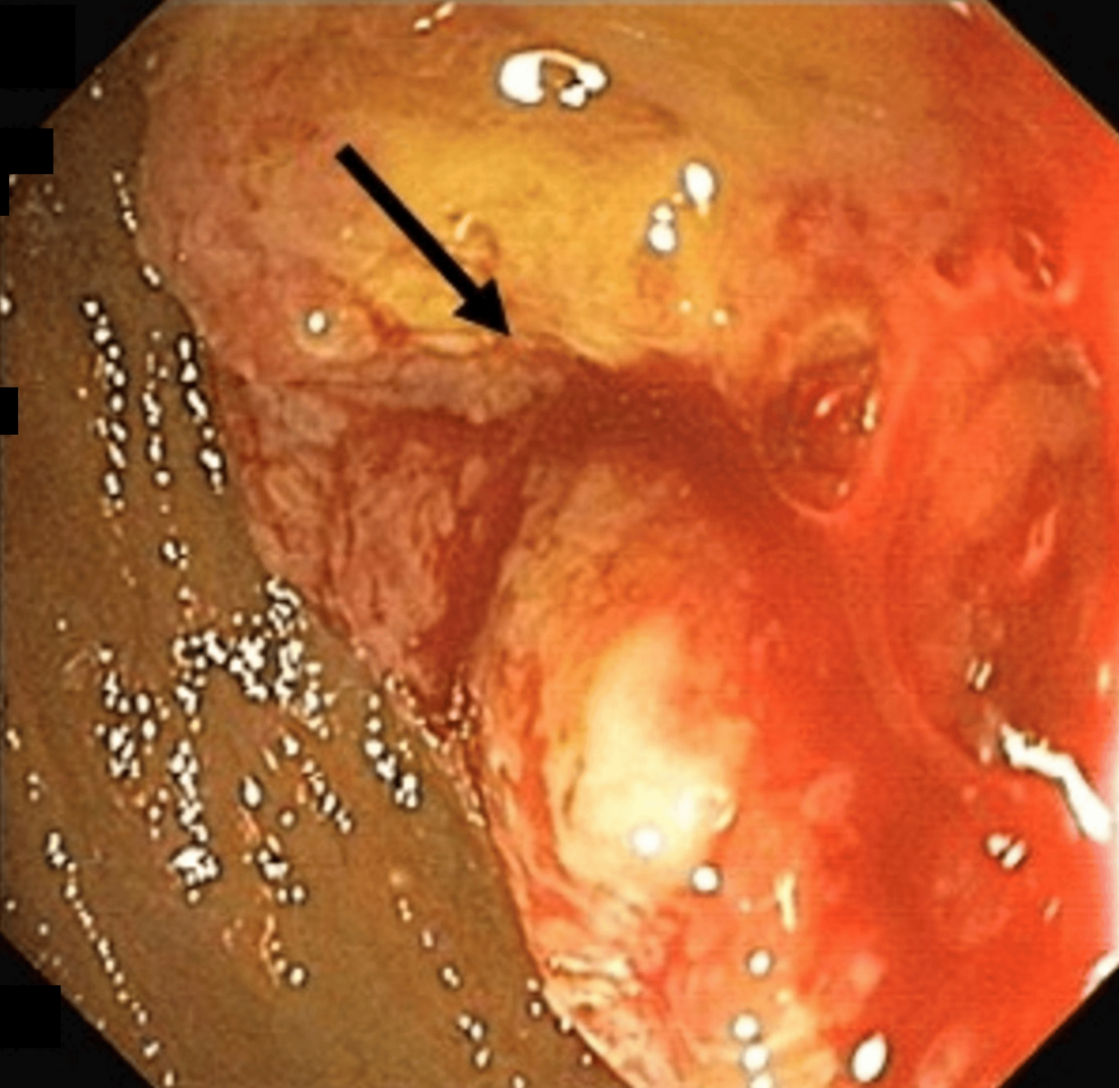
Mass at the junction of the first and second part of the duodenum causing obstruction.

Given the above-mentioned complaints, contrast-enhanced CT of the abdomen with the chest was done that was suggestive of ill-defined, minimally heterogeneously enhancing mass in the periampullary region causing abrupt cut off of the terminal common bile duct and pancreatic duct causing upstream dilatation of the common bile duct, common hepatic duct, bilateral intrahepatic biliary radicle, and infiltrating adjacent duodenal ampulla and the second part of the duodenum causing luminal narrowing with upstream dilatation of the first part of duodenum and stomach with mildly enlarged retroperitoneal and paraaortic lymph nodes. Upper gastrointestinal endoscopy showed an ulcero-proliferative growth in the second part of the duodenum causing near-total narrowing wherein the adult endoscope was not negotiable beyond the obstruction, leading to gastric outlet obstruction (Figure 1).

The patient was planned for endoscopic retrograde cholangiopancreatography, but the procedure was abandoned because of the periampullary mass causing duodenal obstruction and non-passage of the endoscope. Hence, to relieve jaundice, the patient underwent percutaneous transhepatic biliary drainage along with internal metal stenting because of biliary stricture, and a stent measuring 10 × 100 mm was placed. The bilirubin levels started to decrease post-drainage. Two days after the initial procedure, the patient was planned for enteral stenting for the gastric outlet obstruction.

**Figure 2:**
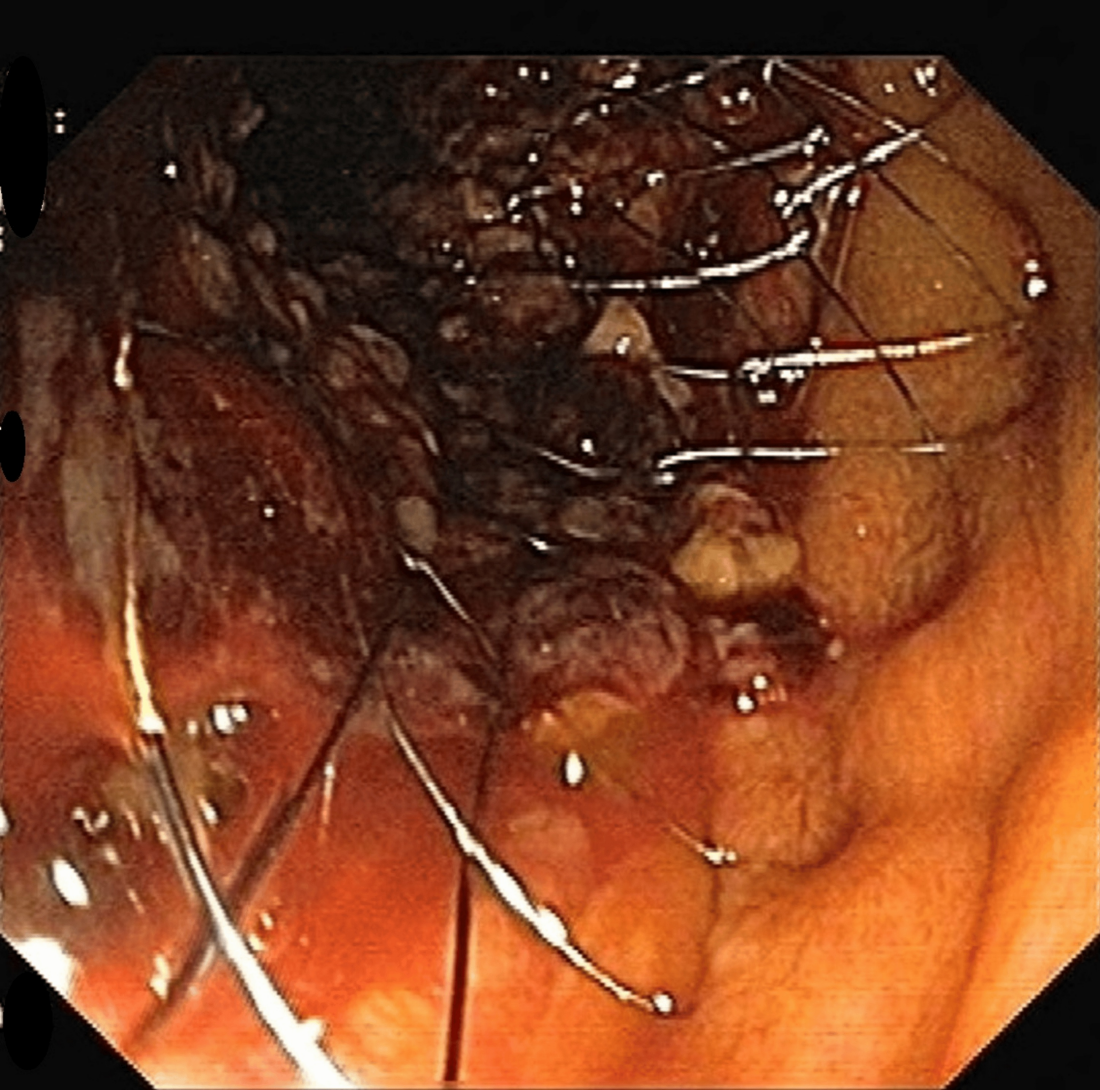
An enteral stent (self-eluting metal stent, fully uncovered size 14 cm × 18 mm) placed at the junction of the first and second part of the duodenum.

The external drain was removed 48 hours post-procedure as there was no collection in the drain. Antibiotics were continued. Three days after the first procedure, an enteral stent (self-eluting metal stent, fully uncovered measuring 14 cm × 18 mm) was placed endoscopically at the junction of the first and the second part of the duodenum for the relief of obstruction caused by the mass. The position of the stent was confirmed both endoscopically and fluoroscopically (Figure 2).

Both the procedures were uneventful. The patient was monitored for complications post-procedure. He was started on an oral diet and was feeling symptomatically better without any complaints. The patient was discharged and advised for follow-up and chemotherapy for further management of the disease.

## Discussion

A heterogeneous group of neoplasms arising from the head of the pancreas, the distal common bile duct, and the duodenum are defined as periampullary carcinomas [4]. The choice between different treatment options available depends on tumor location, disease stage, and the goals of treatment, along with treatment effectiveness and morbidity and mortality [4,5]. Interventional endoscopy or surgical procedures are the current treatment modalities for biliary and duodenal complications arising from pancreatic adenocarcinoma [6].

The goal of treating these complications is to achieve the most complete and long-lasting absence of symptoms possible while minimizing the negative impact of the intervention on morbidity and mortality [6,7]. Placement of the prosthesis may be hindered due to challenges in accessing the papilla (such as previous surgery or associated duodenal stricture), or difficulties in cannulating the bile duct or advancing a guidewire through the stenosis [8].

In case of failure, alternatives include attempting a second endoscopic retrograde cholangiopancreatography catheterization, performing decompression via a percutaneous transhepatic route, or using endoscopic ultrasound guidance [9,10]. Symptomatic duodenal stenosis or obstruction due to tumor invasion can also occur in patients with a biliary stent in situ. Once it is confirmed that the biliary stent is functioning properly, or after replacing an occluded prosthesis, a duodenal stent can usually be placed without any difficulty. Duodenal stricture is treated endoscopically by the insertion of an uncovered self-expanding metallic stent into the duodenal lumen through an endoscope with a large working channel [10]. A duodenal stent placement must always be done post-biliary stent placement [10,11].

## Conclusions

The prompt initiation of endoscopic procedures is crucial for the palliative treatment of biliary and duodenal complications. Biliary stent drainage stands out for its remarkable effectiveness and low morbidity. Additionally, this approach has the potential to alleviate symptoms associated with duodenal obstruction.
